# Patrol Detection for Replica Attacks on Wireless Sensor Networks

**DOI:** 10.3390/s110302496

**Published:** 2011-02-28

**Authors:** Liang-Min Wang, Yang Shi

**Affiliations:** 1 School of Computer Science and Communication Engineering, Jiangsu University, Zhenjiang, 212013, China; 2 School of Electronic and Optical Engineering, Nanjing University of Science and Technology, Nanjing, 210094, China; E-Mail: shw@ujs.edu.cn

**Keywords:** wireless sensor networks, security, replica attack, mobile nodes

## Abstract

Replica attack is a critical concern in the security of wireless sensor networks. We employ mobile nodes as patrollers to detect replicas distributed in different zones in a network, in which a basic patrol detection protocol and two detection algorithms for stationary and mobile modes are presented. Then we perform security analysis to discuss the defense strategies against the possible attacks on the proposed detection protocol. Moreover, we show the advantages of the proposed protocol by discussing and comparing the communication cost and detection probability with some existing methods.

## 1. Introduction

Wireless sensor networks are usually deployed in hostile environments for their unattended nature which makes nodes in the network dangerous to be captured by an adversary. The adversary can compromise the captured nodes and obtain all the secrets of the nodes, replicate the compromised nodes to get many replicas with the same node identity. Then she can launch an insidious attack with these “legitimate” nodes.

The compromised node and its replicas can join the network and act as any benign nodes. This is very harmful to the network. As discussed in references [1–3], many detection methods work well to detect the compromised node under the assumption that the benign nodes are in majority in global and local areas, but they didn’t focus on replica attacks, in which the adversary has many malicious replicas, and the assumption of “benign nodes in majority” has thus failed, so we should exclude the replicas before using these compromised node detection methods.

## 2. Related Work and Network Assumptions

### 2.1. Related Work

After Parno, Perrig *et al*. [4] pointed out the concept of replica attack, some detection methods were proposed, such as centralized detection, local detection, and distributed detection. In general, centralized methods will bring out the problem of single point failure, and many communications are converged in the neighborhood of the central node. Local detection doesn’t deal with the replicas deployed in different zones and the communication is too high in the distributed detection. Parno, Perrig *et al.* present randomized multicast and line-selected multicast which use some witness nodes to replace the whole network detection and ensure the detection probability by the birthday paradox theory. Ho *et al.* [5] further decrease the communication cost by using group deployment knowledge.

Ho *et al.* [6] also present a SPRT method for replica detection in mobile sensor networks, in which all sensors are mobile. Pietro, Oligeri *et al.* [7] consider another type of mobile sensor network in which mobile sinks visit stationary sensors and collect the data once in each round. In this letter, we use mobile nodes acting as the mobile sink described in [7] to patrol the stationary sensors and detect the replicas. This likes the policeman in the real society scenario where he patrols the streets to find the bad person which is more efficient than all the citizen checking and report their neighbors.

### 2.2. Network Assumptions

In our network, there are two types of nodes: mobile nodes serving as patrollers and sensor nodes, which we also call ordinary or stationary nodes. Mobile sensor devices are more powerful than stationary ones in terms of battery power, storage and communication band. The mobile nodes are also able to obtain their location information. The sensors organize a two-dimension stationary sensor network where the locations of sensors do not change after deployment.

We assume that all direct communication links between nodes are bidirectional. Every node has a unique ID in the network which is assigned by the network operator before deployment. An identity-based public key scheme and time synchronization system are employed for the nodes and network as the most common attack detection scheme [4,5]. We also assume there is a maximum speed of the mobile nodes in this system as Ho *et al.* [5]. This maximum speed assumption can be used to identify the replicas of mobile nodes if they move faster than the speed limitation.

The adversary has the ability to compromise a limited number of nodes, fully control the compromised node, and produce many replicas of compromised nodes to enlarge the attack ability. We assume that the adversary can’t capture enough nodes to have a significant influence on the network, but may fully control the whole network by replicating many replicas. We also assume that the adversary can’t create new IDs. Thus the goal of this paper is finding and revoking all the replicas with the same ID to ensure the security of the network.

## 3. Patrol-based Replica Detection Protocol

We will detect the replicas by the assumptions presented in Section 2. If two or more sensors in different locations have a same ID, then all the nodes with the ID will be regarded as compromised node or its replicas. Also, if a mobile node moves with a speed higher than the denoted maximum speed, it will be regarded as a replica attack.

### 3.1. Basic Patrol Protocol

The mobile nodes patrol the networks and send their claim messages to sensors. The sensors should get their secret material from the patroller at the proceeding round, or else, it will be excluded from the network in next round.

In the first round, the networks should be initialized. We assume that there are no any attacks at the initial round as in most of the literature [4]. Each node will be patrolled by at least two mobile nodes. After receiving the location messages, the stationary node *N* takes the mobile nodes who patrolled him as the anchor nodes, then using some localization algorithms, such as presented in literature[8], to obtain their location (*x_N_*, *y_N_*), and save (*x_N_*, *y_N_*) as his own location L*_N_*.

**Figure 1.**
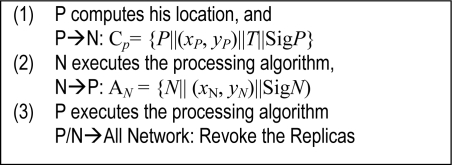
Basic frame patrol detection protocol.

After the initial round, each round is divided into some intervals. In each interval, a patroller will move to a zone to broadcast its claim message. Then the stationary nodes will communicate with a mobile patroller by using the patrol detection protocol as shown in Figure 1 in every round.

As shown in Figure 1, when a mobile patrol node *P* moves to a new zone, it first discovers its location (*x_P_*, *y_P_*) and then broadcasts its patrol claim C*_P_* = {*P*‖(*x_P_*, *y_P_*)‖*T*‖Sig*P*}, where *T* is the claim sent time, Sig*P* is the signature generated by node *P*’s private key K_S_(*P*). In fact, we usually have :

(1) $$\text{Sig}P\;={\left\{\left(x_{P},\;y_{P}\right)\|T\right\}}_{K_{S}(P)}$$

Upon receiving C*_P_*, every neighboring node *N* checks whether *T* is valid or not. If:

|T′−T|>δ+ɛ

where *T'* is the claim receipt time at *N*, *δ* is the estimated transmission delay of claim and, *ɛ* is an acceptable error of the time synchronization system (for ease of exposition and without loss of generality, we use the same symbol *ɛ* in this letter to denote the acceptable errors of all aspects of the networks). Then node *N* will ignore the request. Otherwise, *N* will compute the distance *d’* between his own position (*x_N_*, *y_N_*) and the patroller’s claimed position (*x_P_*, *y_P_*), and compute the relative distance *d* from the received signal power. Then *N* will compare *d* with *d’*. If the difference between the two values exceeds the system accepted error *ɛ*, the node will broadcast a surveillance message S*_N_* = {*N*||*P* ||(*x_N_*, *y_N_*) *||* Sig*N* ||Sig*P*} to report a fault, where Sig*P* is forwarded from *P*’s claim. If the difference is acceptable, it sends A*_N_* = {*N*|| (*x*_N_, *y_N_*)||Sig*N*) to *P*, then save and forwards *P*’s claim to the patroller in the next round with probability *p*.

After collecting the answer message A*_N_*, *P* will check the location of node *N*, and if the distance is larger than the signal range, it ignores the wrong message. Otherwise, *P* checks the *ID* of the answer message by using the security assumption “A benign *ID* only has one location”. Then it saves the answer from the benign node in a white list, saves the replica node’s *ID* in a blacklist, and revokes the replicas’ *ID* by refusing to distribute secrete material and broadcasting its two answer messages to other mobiles nodes. Then *P* will move to other location to send his patrol claim in another interval. After a round, it collects all the saved information of the white and blacklists to the user when collecting the sensing data.

### 3.2. Replicas Detection

In our network model, there are two types of nodes: patrol nodes and ordinary sensors. So there are two kinds of replica detection algorithms.

*Replica Node Detection:* In our network assumption, each sensor node has a unique *ID* and is static after it is deployed. Under the security assumption “A benign *ID* only has one location”, we detect replicas by using patrol nodes to seek for the *ID* in more than one location. If the replicas are deployed in a zone where a patrol node collects their answer message in a patrol interval, then the patroller can revoke them immediately after he receives the second answer and the distance between the two location exceeds *ɛ*. Else if the replicas’ answers are collected by different patrol nodes, then they will be found by the base station or by exchange messages of patrollers after a round. After receiving A*_N_*, P executes the following Node Replica Detection Algorithm.

*Replica Patroller Detection:* If the adversary compromises and replicates the patrol node, then the detection assumption for the static sensor nodes will not work, because the benign mobile patrol node is treated as replica due to the continuous change in locations.

Fortunately, mobility provides us with some clues to help resolve the mobile replica detection problem. Firstly, a benign mobile patroller will wait for the answer message after he reaches a new position and sends his claim in time *T*., so there is a static period *Interval* after the patrol broadcasts his claim. Accordingly, if the patroller node moves and changes its position in time (*T*, *T* + *Interval*), then it is highly likely that at least two nodes with the same identity are present in the networks. Further, the mobile patroller should never move faster than the system-configured maximum speed *V*_max_. As a result, we use the fact that an uncompromised patroller should never move at speeds in excess of *V*_max_ and satisfies formula (1) as following:

(2) $$\left|\frac{\left\|L_{1}-L_{2}\right\|}{\|T_{1}-T_{2}\|-\mathrm{interval}}\right|\leq V_{\text{max}}$$

where *L_i_*, *i* = 1, 2, are the location in time *T_i_* respectively, and the (*L_i_*, *T_i_*) are refined from *P*’s claims forwarded by the monitor sensor nodes in the patrol protocol.

After receiving the patrol claim C*_P_* from *P*, the ordinary node executes following operations shown as the pseudo-code to detect patrol replicas.

**Figure 3.**
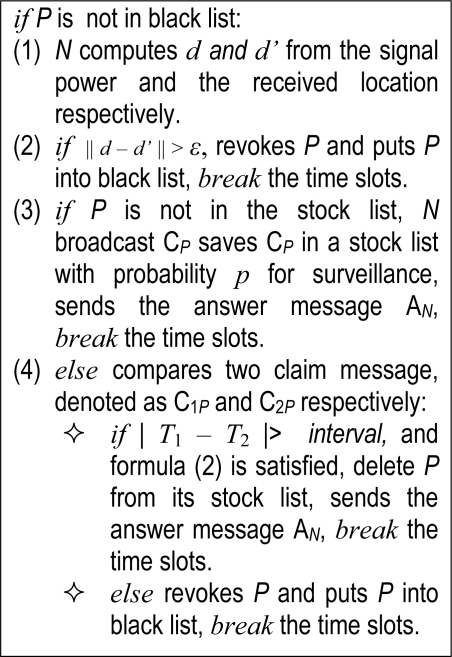
Detection algorithm of patroller replica.

In the algorithm shown in Figure 3, the sensors broadcast C*_P_* with probability *p* as surveillance. This measure provides evidence for mobile replica detection, and the probability *p* decreases network traffic.

## 4. Security and Performance

### 4.1. Security Analysis

The proposed schemes should perform replicas dtection in a secure manner. Let us discuss attacks that might be launched by the attacker and the defense strategies against such attacks in our protocol. Firstly, a malicious sensor may attempt to forge a claim for defaming the patroller. However, there is a signature of *P* in C*_P_*. The malicious node cannot get a fresh *P*’s signature in a forge time *T*, because the time *T* is encrypted by the private key of *P* in Sig*P* defined in formula (1). The malicious node cannot forge a location too. So the Sig*P* present a binding of time and location, which provides the integrity and freshness of the claim message.

Similarly, a malicious patroller will try to revoke good nodes as a replica. If *P* revoke a node *N*, it is required to forward *N*’s answer message A*_N_* = {*N*|| (*x*_N_, *y_N_*) ||*T*|| Sig*N*) from two different place in time *T*. It is difficult to forge *N*’s fresh signature in position (*x*_N_, *y_N_*).

Moreover, the adversary cannot gain much benefit from collusion of malicious nodes and patroller. For example, the adversary will deploy many replicas in the zone of a malicious patroller. But the malicious patroller cannot give a new *ID* to the replica nodes and the zone will be patrolled by another patrol node in next round. Then the benefit is that the replica nodes will not be revoked in a round. But the high density of the replicas will help to be found in next round, and it is harmful to hide the malicious patroller. If we require the sensor nodes to show their admission by binding the patroller’s Signature and its own position with the transmitting message in the run time, then its execution will be restricted further.

Finally, if the multiple replicas of a single node form a physically close group and they can answer all claims with the same location, then it will not be detected by the patrol protocol. But this group strategy substantially limits the region affected by the replicas and thus the attacker will not gain much benefit from using the replicas in the limited region. For example, in a false data injection attack, it would be easy to ensure that only one of the replicas’ data values at a time is accepted by the data aggregators. Similarly, in network application protocols, only one of the replicas’ input values at a time would be taken by their neighbors. In this sense, multiple nodes with the same ID would not have more influence in a region than a single node.

### 4.2. Performance Analysis

We deploy *m* mobile nodes and *n* sensor nodes in a field, and we divide the deployment field into *k* claim zones. Table.1 gives the symbols and their notations.

Now we discuss the performance of our detection protocol with these parameters. In our methods, we add the mobile nodes to an existed static sensor network. If the network has a base station, then we use the convenience from the base station. If there is no base station, then the patrols should contact to exchange the detected information. At first, we consider the scenario that the network has a base station. As the trusted centre, base station can arrange the mobile nodes to patrol the nodes. If there are $\left\lfloor \frac{k}{m}-1\right\rfloor +1$ *interval*s in a *round*, then we can set each zone to be patrol at least once at a round. That is to say, the nodes of *m* zones receive and answer message at each interval. The whole communications of the network are $\left(\left\lfloor \frac{k}{m}-1\right\rfloor +1\right)\times \left(\frac{n}{k}\times m\right)\approx n$. As introduced in reference [4], the communication of centralized detection is $O\left(n\sqrt{n}\right)$, our method is much better than that. In fact, we have hierarchy network architecture in this case. There are three layers: a base station, *m* mobile nodes serve as sink, and *n* sensor nodes. Now we consider the communication cost of local detection of hierarchy network with *m* sink nodes. The detection costs within a zone are $\frac{n}{m}$.

The average cost of a sink sending the message to the base station are

$$\sqrt{\frac{n}{m}}\times \sqrt{m}=\sqrt{n}$$

Then the whole cost are

$$m\times \left(\frac{n}{m}+\sqrt{n}\right)\approx O(m\cdot n)$$

It is also higher than our method. Align better all these equations.

Further, we consider the scenario without a base station in the network. If we set ([*k*/*m*–1]+1) *interval*s in a *round* as the case with a base station in the network, then we can’t detect the replicas among different zones though all the nodes are patrolled at a round. The naïve thinking is that each pair of mobile nodes communicates and exchanges all the answer messages at each round. The communications are

$${2C}_{k}^{2}=k(k-1)$$

The cost is too high with the consideration of the exchanged messages.

In fact, it is difficult that each zone will be visited once by a mobile node in this case. The mobile nodes should cost more communication to set the global arrangements of the patrol process. In the following, we set the mobile nodes without global awareness move as the random zone model as the random waypoint model defined by [7], in which each patrol randomly choose a destination zone at each interval. We assume that a *round* has *x interval*s, then the whole communications are (x*n*m/*k*).

Now we discuss the detection probabilities of a node with *r* replicas: *N*_1_, *N*_2_, … *N_r_*. Each replica has $\frac{m}{k}$ probability to be patrolled at an interval, and it has *x* *m/*k* chances to be visit by mobile nodes. Following the standard derivation of the *birthday paradox*, the probability *P*_1_ that *x* *m/*k* mobile nodes patrol the zone located by *N*_1_ does not patrol the *N*_2_’s zone is given by:

$$P_{1}={\left(1-\frac{m\cdot x}{k^{2}}\right)}^{\frac{m\cdot x}{k}}$$

Similarly, the probability *P_i_* that *i*·(*m*·*x*/*k*) mobile nodes that patrol the zones located by one replica of { *N*_1_, *N*_2_, …, *N_i_*} does not patrol the *N_i_*_+1_’s zone is given by:

$$P_{i}={\left(1-\frac{i\cdot m\cdot x}{k^{2}}\right)}^{\frac{m\cdot x}{k}}$$

Thus, the probability *P*_none_ that no two zones with any nodes in {*N*_1_, *N*_2_, … *N_r_*} are patrolled by a mobile nodes is:

$$\begin{array}{lll} P_{\text{none}} & = & \prod_{i=1}^{r-1}{\left(1-\frac{i\cdot m\cdot x}{k^{2}}\right)}^{\frac{m\cdot x}{k}}\\ & \leq & \prod_{i=1}^{r-1}e^{\frac{i\cdot m^{2}\cdot x^{2}}{k^{3}}}\\ & = & e^{\sum_{i=1}^{r-1}\frac{i\cdot m^{2}\cdot x^{2}}{k^{3}}}\\ & = & e^{-\frac{m^{2}\cdot x^{2}\cdot r\cdot (r-1)}{{2k}^{3}}} \end{array}$$

So the detection probability is:

(3) $$\begin{array}{lll} P_{\text{detection}} & \geq & 1-e^{-\frac{m^{2}\cdot x^{2}\cdot r\cdot (r-1)}{{2k}^{3}}} \end{array}$$

If we have *m* = *k* and *x* = *k*^1/2^, *P*_detection_ is greater than 63% in formula (3) when *r* = 2. And *P*_detection_ will be greater than 95% if *r* = 3. In this case, the communication cost are (n*k^1/2^), which is *O*(*n*) if *k* is set independent of *n*.

We show the communication cost of existing work in Table 2. Contrasted with the context, our method is much less than *O*(*n*^2^) of Randomized Multicast in communication cost with the same detection performance, and shows good detection performance over Line-Selected Multicast method with *O*(n*k^1/2^) communication cost over its *O*(n*n^1/2^), in which *k* is much smaller than *n*.

## 5. Conclusions

We use mobile nodes as patrollers to detect replica nodes in wireless sensor networks, and present a patrol detection protocol and related algorithms. Contrasted with existing work, our detection protocol gets best detection performance with similar communication cost and the lowest communication cost with similar detection rates. That is to say, the use of mobile nodes can save the energy of static nodes and prolong the lifetime of the whole network.

## Figures and Tables

**Figure 2.**
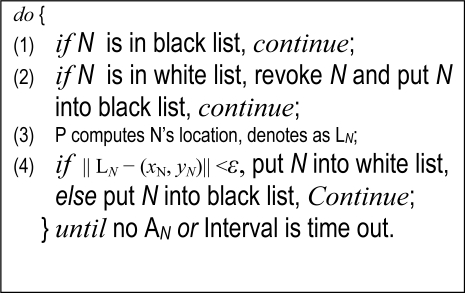
Detection algorithm of node replica.

**Table 1.** Some Notations used in this section.

**Symbols**	**Notation**

Interval	Time period for patrolling a zone.
Round	Time period for the user to collect data
*k*	Total number of zones
*n*	Total number of sensors
*m*	Number of mobile nodes
*r*	Replicas number of a compromised node

**Table 2.** Communication cost. Scale and align equations.

	**Detection Methods**	**Communications**

With Base station	Centralized Detection	*O*(n*n^1/2^)
	Hierarchy Detection	*O*(n*k)
	SPRT for mobile nodes [5]	*O*(n*n^1/2^)
	Our method	*O*(*n*)

Without Base Station	Randomized Multicast [4]	*O*(*n^2^*)
	Line-Selected Multicast [4]	*O*(n*n^1/2^)
	Group deployment [6]	Determined by Deployment Accuracy
	Our method	*O*(n*k^1/2^)
